# Establishment and application value of a novel prescription medication abuse monitoring model for psychiatric hospitals

**DOI:** 10.3389/fpsyt.2022.1082538

**Published:** 2023-01-09

**Authors:** Zhiqiang Du, Ying Jiang, Rongrong Lu, Yuan Shen, Mengmeng Ou, Zhe Wang, Lina Cao, Qin Zhou, Haohao Zhu

**Affiliations:** ^1^The Affiliated Mental Health Center of Jiangnan University, Wuxi Central Rehabilitation Hospital, Wuxi, Jiangsu, China; ^2^Wuxi Institute of Drug Control, Wuxi, Jiangsu, China

**Keywords:** psychiatric hospital, drug concentration monitoring, hospital information system, retrospective analysis, prescription medication abuse monitoring model

## Abstract

**Objective:**

To construct a prescription medication abuse (PMA) monitoring model for psychiatric hospitals and to assess its applicability.

**Methods:**

A PMA monitoring working group was established to guide the formulation of a PMA monitoring system, which included three active real-time monitoring modes and one retrospective analysis monitoring mode. The effect of the established system was analyzed.

**Results:**

In 2021, 35 cases of effective PMA were reported, which was a significant increase compared to two cases identified through passive monitoring mode in the preceding year. Most of the reported cases were based on active real-time monitoring mode. Among them, 21 cases (60.00%) were identified during the diagnosis and treatment of medicine and nursing; 3 cases (8.57%) were reported based on drug concentration detection technology; and 5 cases (14.29%) were reported by the laboratory department during PMA screening. Besides, 6 cases (17.14%) were reported according to the retrospective analysis of the hospital information system. The majority of prescription medication abusers were adolescents under the age of 18 (12 cases, 34.29%). Overall, there were 27 cases of class II psychotropic prescription medications, accounting for 77.14%.

**Conclusion:**

The combined PMA monitoring model can effectively improve the quality of PMA monitoring and provide a basis for the supervision of higher-level regulatory authorities.

## 1. Introduction

Prescription medication abuse (PMA) refers to the repeated and large-scale use of prescription medications with dependence characteristics or dependence potential without medical purposes (1). Prescription medications that are frequently abused include non-pharmaceutical and pharmaceutical preparations, including illicit substances prohibited for medical use and regulated prescription medications. PMA can lead to addiction, and other behavioral disorders, causing severe public health and social problems (2, 3). Although the prescription drug monitoring program conducted in the US has achieved a certain effect (4, 5), the model could not be adopted by China due to the different national conditions. The main reason is the strict policy against PMA in China, which limit the PMA epidemic. In addition, the different prescription networks, pharmacy setups and pharmacist coverage hinder adoption of the application of the prescription drug monitoring programs in China. There are two main methods of PMA monitoring in China: Passive monitoring and active monitoring (6, 7). Although passive monitoring is the main model at present, active monitoring is becoming increasing important. According to the “13th 5-Year” National Drug Safety Plan, by the end of 2020, 100 specialized medical institutions and general medical institutions for mental diseases were selected nationwide as PMA monitoring sentinel points to conduct active PMA monitoring (8).

A study involving in 36 monitoring institutions in 12 provinces and 358 medical staff of 61 medical institutions concluded that more than 83.8% of the participants believed that PMA monitoring should be carried out in psychiatric hospitals (9). Patients abusing psychoactive substances may seek medical treatment after impaired physical and mental health in psychiatric hospitals, thus such institutions can conveniently collect the abuse of psychotropic drugs. Furthermore, such PMA data could be reported to provide a reference to improve the management of psychiatric hospitals and psychiatric drugs.

However, no PMA monitoring system is currently executed in psychiatric hospitals in China. Therefore, establishing a PMA monitoring model for psychiatric hospitals may provide the technical system required for psychotropic prescription medication supervision and facilitate the timely adjustment of national prescription medication supervision policies. As a national PMA monitoring sentinel hospital recognized since 2020, and the passive monitoring model was adopted in 2020. After retrospective analysis of its defects, a PMA monitoring working group was established to guide the formulation of the combined PMA monitoring mode, and its efficacy was evaluated.

## 2. Materials and methods

### 2.1. Establishment of the working group and formulation of the work system

After preliminary investigation of the whole hospital, a team consisting of the pharmacy department, medical department, nursing department, outpatient department, laboratory department, information department, and clinical department, was established in 2021. The PMA monitoring liaison officers were informed about responsibilities and the reporting system. The Clinical Pharmacy Department was specifically responsible for the management of PMA monitoring, and a clinical pharmacist was designated to be responsible for data review, analysis, and reporting.

### 2.2. Determining the diagnostic criteria and monitoring varieties of PMA

The diagnostic criteria of PMA refer to the criteria established by the World Health Organization (10): continuous or intermittent excessive use of prescription medications, which is irrelevant or contradictory to reasonable medication. PMA can include several scenarios: more than prescribed medication was used because they were not getting the appropriate clinical impact on the lower doses; they could be acquiring medications from a source other than a doctor; they could also be injecting/snorting oral medication. For preliminary assessment of PMA monitoring and combined with the prescription medication catalog of our hospital, we systematically sorted out all the monitoring varieties, including 3 categories and 16 kinds (Table 1). Compound paracetamol and amantadine hydrochloride capsules contained paracetamol, amantadine hydrochloride, chlorphenamine maleate, artificial bezoar, and caffeine. Compound paracetamol tablets included paracetamol, aspirin and caffeine. Anmameimin tablets contained paracetamol, pseudoephedrine hydrochloride, dextromethorphan hydrobromide and chlorphenamine maleate.

**TABLE 1:** Categories of PMA monitoring.

Class	Prescription medications
Class II psychotropic drugs	Oxazepam, Zopiclone, Eszopiclone, Zolpidem, Clonazepam, Lorazepam, Alprazolam, Etizolam, Diazepam
Narcotics	Methamphetamine, Heroin, Ketamine, 3,4-methylenedioxy- methamphetamine
Compound preparations containing psychoactive substances	Compound paracetamol and amantadine hydrochloride capsules, Compound paracetamol tablets, Anmameimin tablets

### 2.3. Establishment of the working model for PMA monitoring

#### 2.3.1. Active real-time PMA monitoring mode

**FIGURE 1:**
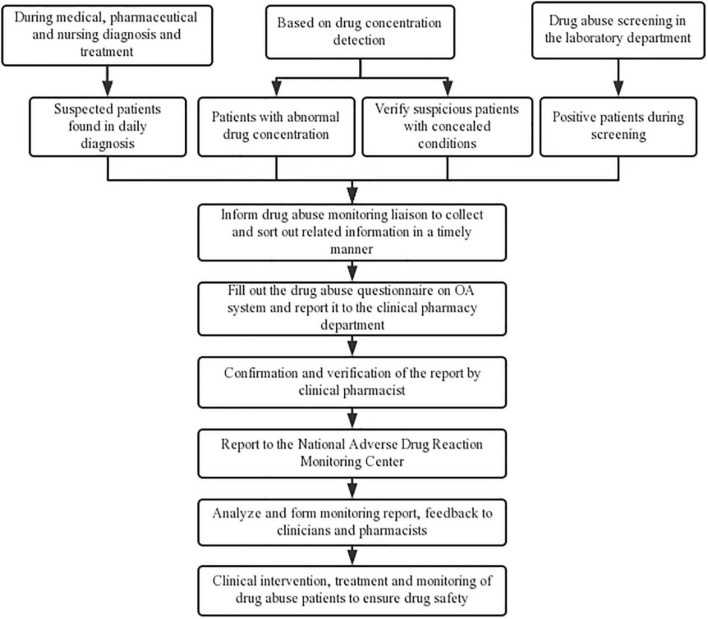
Details of the active real-time PMA monitoring mode.

The active real-time PMA monitoring mode contained three parts (Figure 1). The first was during regular diagnosis and treatment. During daily diagnosis and treatment, consultation and prescription review by doctors, nurses, and pharmacists of outpatients and inpatients, the suspected PMA patients could be detected and reported to the PMA monitoring liaison in a timely manner. After confirmation, patient data were collected to complete a PMA questionnaire for reporting through the OA system. The OA system is tailored to the specific hospital and integrated in the internal management of the hospital, which supports the management of all departments. It could integrate the personnel, business processes, information, organization and office automation technology and equipment into an organic system, and constitutes a unified working platform for the personnel of the hospital. The second was based on prescription medication concentration detection technology. Patients showing abnormally high drug concentrations involved in the monitoring varieties would be found by the pharmacists conducting daily drug concentration monitoring with informed consent. Besides, for suspicious patients with concealment during diagnosis and treatment, drug concentration detection technology was applied to test the concentration of suspicious prescription medications. The third was based on the laboratory PMA screening. As the study was conducted in a psychiatric hospital, all patients had the potential risks of PMA, thus PMA screening was performed on all inpatients and outpatients who visited our hospital with informed consent. Abnormal results were reported by the laboratory department and verified by doctors and pharmacists.

#### 2.3.2. Retrospective PMA monitoring mode

**FIGURE 2:**
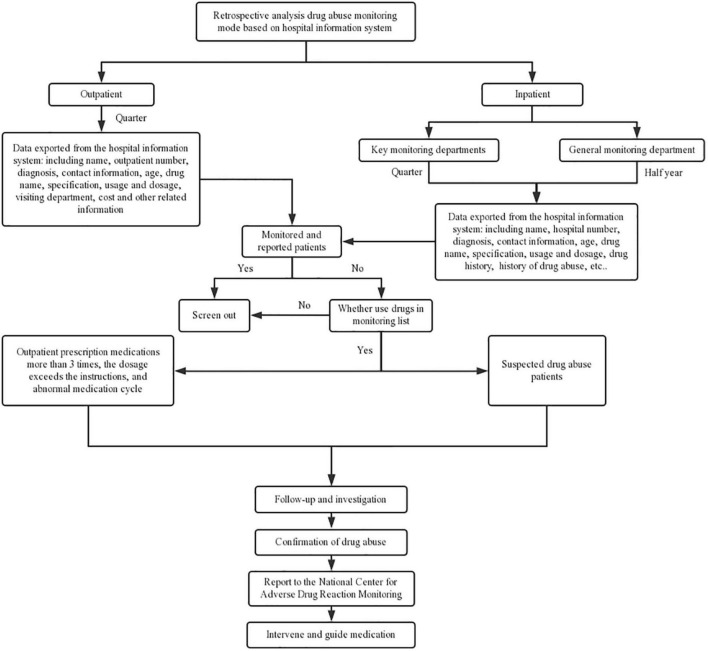
Details of retrospective PMA monitoring mode.

The hospital information department was instructed to cooperate with pharmacists to facilitate the retrospective analysis monitoring mode (Figure 2), which supplemented the active real-time monitoring mode. For outpatients, the hospital information system data were exported every quarter, and the data included basic information such as gender, age, and diagnosis, as well as the number of outpatient prescriptions, prescription medication usage and dosage, prescription medication use days, and prescription medication costs. After excluding the patients who had been reported through active real-time monitoring, the remaining patients with more than three outpatient prescriptions, dosages exceeding the instructions, and abnormal medication cycles were selected for a follow-up to confirm PMA. For hospitalized patients, those departments (psychiatry, clinical psychology, drug dependence, sleep medicine) that used more prescription medications included in the PMA monitoring catalog were examined. Quarterly data on usage, frequency and dosage of prescription medications in the PMA monitoring catalog, and the prescription medication use and abuse history, were used to screen suspected prescription medication abusers. The selected patients were followed up to identify PMA. Those who met the reporting standards were reported in a timely manner, and interventions were conducted to guide rational prescription medication use. For departments that used fewer prescription medications included in the PMA monitoring catalog, the data screening and monitoring of the hospital information system was carried out every 6 months.

The clinical pharmacist was responsible for the review of the report and was subjected to the National Adverse Drug Reaction Monitoring Center after verification and confirmation. The reports were analyzed and fed back to the clinicians and pharmacists to conduct clinical intervention, treatment and monitoring of PMA to ensure medication safety.

### 2.4. Statistical analysis

Descriptive statistics were applied. Data were input into EpiData 3.1 software and statistically analyzed using SPSS 20.0. The counting data were described as the number of cases (composition ratio), and the measurement data were described by x ± s.

## 3. Results

### 3.1. Number of reported cases of PMA

Comparing the combined PMA monitoring mode with the passive monitoring mode in the previous year, the number of effective PMA cases reported in 2021 was 35, which was a marked increase from the 2 cases in the previous year. Among them, 21 cases of PMA were detected through the active real-time monitoring mode during the diagnosis and treatment of medicine, medicine and nursing, accounting for 60.00%; 3 cases of PMA were reported through active real-time monitoring by pharmacists using drug concentration detection, accounting for 8.57%; during PMA screening in the laboratory department, 5 cases of PMA were detected in real time with abnormal results, accounting for 14.29%. The retrospective analysis of PMA in the hospital information system detected 6 cases of PMA, accounting for 17.14% see Table 2.

**TABLE 2:** Numbers of reported cases of PMA under the new PMA monitoring model in 2021.

Monitoring model	Cases	Ratio/%
Active real-time monitoring during medical, pharmaceutical and nursing diagnosis and treatment	21	60.00
Active real-time monitoring by pharmacists using drug concentration detection	3	8.57
Active real-time monitoring during PMA screening in the laboratory	5	14.29
Retrospective analysis monitoring in hospital information system	6	17.14

### 3.2. Basic information of reported patients

Fourteen cases of PMA were males (40.00%), and 21 cases were females (60.00%). The age distribution ranged from 13 to 82 years of age, among which 12 cases were ≤18 years old (34.29%), and all of them abused class II psychotropic drugs to relieve anxiety and depression (Table 3).

**TABLE 3:** Basic information of reported patients in 2021.

Factors	Cases	Ratio/%
**Gender**		
Male	14	40.00
Female	21	60.00
**Age**		
≤18	12	34.29
19–25	6	17.14
25–35	3	8.57
35–45	3	8.57
45–55	6	17.14
55–65	4	11.43
>65	1	2.86

### 3.3. Prescription medication distribution of reported PMA

Thirty-five cases of PMA were mainly classified as class II psychotropic drugs, accounting for 27 cases (77.14%), followed by narcotics and compound preparations containing psychoactive substances, each with 4 cases, accounting for 11.43% (Table 4). Among them, abuse of benzodiazepines was most common, including oxazepam and clonazepam.

**TABLE 4:** Prescription medication distribution of reported PMA.

Prescription medications	Cases	Ratio/%
Class II psychotropic drugs	27	77.14
Oxazepam	7	20.00
Clonazepam	7	20.00
Sulazepam	5	14.29
Zopiclone	3	8.57
Lorazepam	2	5.71
Zolpidem	1	2.86
Diazepam	1	2.86
Nitrazepam	1	2.86
Narcotics	4	11.43
Methamphetamine	2	5.71
Heroin	1	2.86
Ketamine	1	2.86
Compound preparations containing psychoactive substances	4	11.43
Compound paracetamol and amantadine hydrochloride capsules	2	5.71
Compound paracetamol tablets	1	2.86
Anmameimin tablets	1	2.86

## 4. Discussion

The problem of PMA has attracted increasing attention around the world as it causes considerable harm to public health and is an important factor affecting social security (11). PMA monitoring is an indispensable and important task for the state to collect PMA information and to maintain public safety (12, 13). At present, a complete legal and regulatory system for PMA monitoring, a clear organizational framework, functional responsibilities, and personnel funding guarantees are lacking (14). China pays increasing attention to PMA monitoring, and 100 medical institutions nationwide have been selected as PMA monitoring sentinel sites to gradually establish and improve the PMA monitoring legal system, organizational framework, and functional responsibilities and establish relevant standards.

Our hospital is the first psychiatric hospital in China to explore and carry out PMA monitoring in China. This is still at the preliminary stage, and there is no absolute model for reference. Nonetheless, the PMA monitoring model established in this study can help with monitoring efforts of the medical, pharmaceutical, nursing and inspection departments and make up for the possibility through drug concentration detection technology, PMA screening technology, and retrospective analysis of information systems for underreporting problems due to high workload, patient concealment, etc., which can cover almost all suspected patients with PMA in our hospital. The advantages of passive monitoring include simplicity of use and no requirement for additional manpower and material resources, while the disadvantages include the high underreporting rate and single category. Active monitoring can significantly reduce the rate of false positives and increase the initiative of medical, pharmaceutical, nursing, and inspection departments. Among them, after PMA screening, patients who abuse prescription medications can be found in time, tracked in detail, monitored and reported. The reported and tested substances are basically the same. At the same time, drug concentration detection technology, PMA screening technology, and retrospective analysis through information systems can compensate for underreporting, so as to cover almost all suspected cases. This study was funded by Wuxi ADR Monitoring Center, and the corresponding personnel were rewarded according to the number of reported cases (50 yuan/case). Later, the hospital will set up relevant special funds to cover the human costs required for PMA screening. However, active monitoring required more manpower, material resources, and funding. The combined monitoring method may integrate the above advantages, however, there are still many challenges, such as only approximately 11.44% of patients agreeing to PMA screening and few patients were willing to take the initiative to inform doctors about the details of PMA. On the one hand, it is necessary to carry out and improve monitoring work mode and systems on long term and in a continuous and systematic manner. In addition, it is also necessary to trace the source of the abused prescription medications, find the weak links in supervision, and strengthen the supervision of the production, storage and circulation of certain particular prescription medications (5, 15, 16).

Adolescents accounted for the majority of prescription medication abusers, which may be because of increasing societal and academic pressure (17–19). As mental health problems, such as anxiety, depression, and insomnia, are becoming increasingly prominent, more class II psychotropic drugs are dispensed, which leads to PMA among adolescents. Through PMA monitoring work, early warning can be improved, and potential PMA may be monitored, detected, resolved, and prevented. Pharmacists can selectively focus on the class II psychotropic drugs used by adolescents according to the monitoring results to provide pharmaceutical services from the aspects of indications, usage and dosage, adverse reactions, and addiction hazards to strengthen the awareness of adolescents regarding these prescription medications and urge their guardians for better supervision of prescription medications to better ensure the safety, effectiveness and rationality use in adolescent patients.

The PMA monitoring model constructed in this study can effectively link people, information systems and novel pharmaceutical testing technologies to improve the reporting rate and quality of PMA. The application of the established model could significantly reduce the rate of missing reports and timely find out the prescription medication abusers to provide symptomatic treatment, which greatly improves the treatment prognosis and reduces the risk of overdose. At the same time, the model can help relevant departments to strengthen monitoring to reduce crimes in society. Furthermore, as the technologies involved in the model are routine projects carried out by hospitals, no extra costs are needed except additional person-hours, and the model can be implemented in other countries/health systems. However, there are still some shortcomings: no relevant reward mechanism has been established, and the enthusiasm of personnel to participate needs to be improved; insufficient publicity of PMA-related knowledge, resulting in variability of monitoring reports. Subsequent research will further improve the PMA monitoring model and promote its application to improve the monitoring and reporting awareness of hospitals, reduce the rate of underreporting, collect data on PMA in a timely and effective manner, discover problems and risks of PMA, and grasp the types and current characteristics of PMA to provide the basis for the supervision of anesthesia and psychotropic prescription medications for the superior supervision department.

## 5. Conclusion

The combined PMA monitoring model established can effectively improve the reporting rate of PMA. It has been proven by practice that this mode can play a leading role in the monitoring of clinical departments and make up for the problem of missing reports caused by the busy work of clinical departments by promoting exchanges and cooperation between medical and pharmaceutical departments.

## Data availability statement

The original contributions presented in this study are included in the article/supplementary material, further inquiries can be directed to the corresponding authors.

## Ethics statement

Ethical review and approval was not required for the study on human participants in accordance with the local legislation and institutional requirements. Written informed consent for participation was not required for this study in accordance with the national legislation and the institutional requirements.

## Author contributions

ZD and HZ conceived the study. ZD, RL, YS, MO, ZW, and QZ collected the report. YJ and LC wrote the manuscript. QZ and HZ edited and revised the manuscript. All authors contributed to the article and approved the submitted version.
